# X-Ray Spectroscopy of Ultra-Thin Oxide/Oxide Heteroepitaxial Films: A Case Study of Single-Nanometer VO_2_/TiO_2_

**DOI:** 10.3390/ma8085255

**Published:** 2015-08-21

**Authors:** Nicholas F. Quackenbush, Hanjong Paik, Joseph C. Woicik, Dario A. Arena, Darrell G. Schlom, Louis F. J. Piper

**Affiliations:** 1 Department of Physics, Applied Physics and Astronomy, Binghamton University, Binghamton, NY 13902, USA; E-Mail: quackenbush@binghamton.edu; 2 Department of Materials Science and Engineering, Cornell University, Ithaca, NY 14853, USA; E-Mails: hp277@cornell.edu (H.P.); schlom@cornell.edu (D.G.S.); 3 Materials Science and Engineering Laboratory, National Institute of Standards and Technology, Gaithersburg, MD 20899, USA; E-Mail: woicik@bnl.gov; 4 National Synchrotron Light Source-II, Brookhaven National Laboratory, Upton, NY 11973, USA; E-Mail: darena@bnl.gov; 5 Kavli Institute at Cornell for Nanoscale Science, Ithaca, NY 14853, USA

**Keywords:** heteroepitaxial systems and interfaces, rational design of nanoscale materials, structure-function relationship

## Abstract

Epitaxial ultra-thin oxide films can support large percent level strains well beyond their bulk counterparts, thereby enabling strain-engineering in oxides that can tailor various phenomena. At these reduced dimensions (typically < 10 nm), contributions from the substrate can dwarf the signal from the epilayer, making it difficult to distinguish the properties of the epilayer from the bulk. This is especially true for oxide on oxide systems. Here, we have employed a combination of hard X-ray photoelectron spectroscopy (HAXPES) and angular soft X-ray absorption spectroscopy (XAS) to study epitaxial VO_2_/TiO_2_ (100) films ranging from 7.5 to 1 nm. We observe a low-temperature (300 K) insulating phase with evidence of vanadium-vanadium (V-V) dimers and a high-temperature (400 K) metallic phase absent of V-V dimers irrespective of film thickness. Our results confirm that the metal insulator transition can exist at atomic dimensions and that biaxial strain can still be used to control the temperature of its transition when the interfaces are atomically sharp. More generally, our case study highlights the benefits of using non-destructive XAS and HAXPES to extract out information regarding the interfacial quality of the epilayers and spectroscopic signatures associated with exotic phenomena at these dimensions.

## 1. Introduction

With advancements in high quality thin film growth of strongly-correlated oxides, such as molecular beam epitaxy (MBE) and puled-laser deposition, there is rejuvenated interest in oxide electronics [1,2,3]. Transition metal oxides in particular are an exciting class of materials that may lend their properties to future devices. Their partially-filled *n*d-states are comparable in energy to the valence (*n* + 1)s-states, but due to the greater angular momentum, they do not range as far from the nucleus. This causes their behavior to be intermediate between localized and itinerant, while the electrons are highly interacting, often giving rise to collective phase transitions that can be activated under various external stimuli [2,4,5]. This can often result in exciting phenomena, including superconductivity, colossal magneto-resistance and metal-insulator transitions. Furthermore, coherent oxide/oxide heteroepitaxial film growth provides the opportunity to tailor these properties via biaxial strain [6]. In fact, ultra-thin films can support large percent level strains and are robust over millions of cycles through electronic-structural phase changes, both of which are known to cause fracturing in their bulk counterparts [7,8,9]. This makes this class of materials of great interest from both fundamental and technological perspectives.

The metal insulator transition (MIT) is perhaps the most exciting emergent phenomenon that may be utilized in next-generation oxide electronics, such as non-volatile memory [10], Mott field effect transistors [11,12], smart window coatings [13,14] and thermal and chemical sensors [15,16,17]. Widely considered amongst the most suitable candidates for these technologies is vanadium dioxide [9,12,18,19]. VO_2_ exhibits an abrupt and ultrafast MIT near room temperature that can be triggered by small, thermal [20,21], chemical [22], mechanical [23] and electrical [24,25] perturbations. By applying tensile/compressive epitaxial strain along the rutile c-axis, the thermally-induced transition temperature (T*_MIT_*) can be tuned over a range of more than ±40 °C [26], although the complex physics, especially near T*_MIT_*, is still poorly understood [27]. The exact mechanism of the transition remains under intense debate, while both theory and experiments point to cooperation between a Peierls-type and a Mott-type transition [28,29,30]. In fact, it has been suggested that epitaxial strain can even shift the MIT mechanism from more Peierls-like to more Mott-like [31]. There are also at least three distinct structural phases near T*_MIT_* under varying strains, with a solid state triple point at 65 °C and zero strain [32,33,34].

The use of X-ray photoelectron spectroscopy (PES) and oxygen K-edge X-ray absorption spectroscopy (XAS) to examine the occupied and unoccupied electronic structure of transition metal oxides has long been established [35,36,37,38,39], and they remain important tools today for epitaxial transition metal oxide films [40]. The high temperature metallic and low temperature insulating phases of VO_2_ can be easily distinguished using X-ray photoelectron spectroscopy, either from the appearance of a Fermi edge [29] or from the core-level line-shapes [41]. Meanwhile, soft XAS is highly sensitive to the vanadium-vanadium (V-V) dimers because of the pronounced orbital dichroism of the $d_{x^{2}-y^{2}}$ orbital (also referred to as $d_{\left|\right|}$*), associated with the dimers. The resultant orbital dichroism is obvious in both the O K- and the V $L_{3,2}$-edges [30,38]. These methods are ideal for cleaved surfaces of bulk crystals, but thin films require more considerations. In the case of VO_2_, the MIT is highly sensitive to the quality of the interface [42,43,44]. Meanwhile, the surface preparation of atomically-clean VO_2_ for PES measurements is non-trivial.

Hard X-ray photoelectron spectroscopy (HAXPES) is becoming an increasingly important technique for studying the bulk properties of solids, due to the high kinetic energy of the emitted photoelectrons combined with the exceedingly high photon flux of modern synchrotron sources. Traditional PES of transition metal oxides is often hindered by the need for clean surfaces. Even careful surface preparation techniques, such as gentle ion bombardment or low temperature annealing in an O_2_ partial pressure, can often reduce the transition metals to lower oxidation states at the surface, e.g., Mn^2+^ of manganites [45,46]. This is a severe problem for VO_2_, where even the partial reduction of vanadium at the surface can result in the formation of various V*_x_*O*_y_* metastable phases that can still display metal-insulator transitions. These surface reduction issues can be circumvented by use of HAXPES, which increases the effective probing depth and reduces the sensitivity to attenuating carbonate and hydroxyl overlayers compared to traditional PES [47,48]. The orbital and elemental sensitivity of XAS already reduces the need for atomically-clean surfaces. The ability to combine O K-edge XAS and HAXPES of *ex situ* samples free of surface reduction makes it very appealing for studying transition metal oxides [40,49], including VO_2_ [44]. Moreover, the effective probing depth of HAXPES enables one to study the electronic and chemical properties of buried interfaces for ultra-thin epitaxial films [50,51]. However, the inherent properties of ultra-thin oxide/oxide heteroepitaxial films can create some difficulties for both experiment and analysis. The small volume of the oxide film combined with spectral contamination from the oxide substrate can make expounding useful information difficult. Additionally, at these dimensions, one needs to consider carefully both the film surface, as well as the interface quality.

Here, we describe a methodology in which HAXPES and XAS can be utilized to study ultra-thin oxide/oxide heteroepitaxial films with phase changing behavior. We present HAXPES of the core-levels and valence band and XAS at the O K-edge, each above and below the thermally-induced MIT in VO_2_/TiO_2_ (100) epitaxial films. With this approach, we demonstrate that the MIT can be observed in films as thin as 1 nm.

## 2. Experimental Section

Epitaxial VO_2_ thin films were grown on rutile TiO_2_ (100) single-crystal substrates by reactive MBE in a Veeco GEN10. Substrates were prepared by etching and annealing to have clean and well-defined step and terrace microstructured surfaces [52,53]. Vanadium metal and distilled ozone were co-deposited onto the substrate held at 250 °C under a distilled ozone background pressure of 1.0 × 10^−6^ Torr. Sample thicknesses were estimated from the atomic layer growth rates as determined by monitoring the oscillations in reflection high energy electron diffraction (RHEED). A detailed account of a similar sample growth on TiO_2_ (001) substrates is reported elsewhere. [54] Temperature-dependent electrical transport was measured using the standard van der Pauw 4-point method, where the sample was contacted with gold wires and silver paint. These studies confirmed that our films display MITs for film thicknesses of 7.5–2.5 nm [55]. Our TEM studies confirmed an atomically sharp interface for the 7.5 nm sample, but this constitutes a separate work.

The HAXPES measurements were performed at the National Institute of Standards and Technology (NIST) bending magnet beamline X24a at the National Synchrotron Light Source (NSLS) at Brookhaven National Laboratory. Measurements were performed using a photon energy of h*ν* = 4 keV and a pass energy of 500 eV, with a corresponding Gaussian instrumental broadening of 0.45 eV. The binding energy axes were referenced to both the Au 4f_7/2_ and Fermi edge of a Au foil in electrical contact with the film. The take-off angle was 85° to maximize bulk sensitivity. HAXPES was performed on samples at room temperature and at an elevated temperature of 375 K.

XAS of the O K-edge was measured in total electron yield (TEY) mode at beamline U4b of the NSLS by measuring the sample drain current and was normalized by the current from a clean gold mesh placed in the incoming beam to compensate for fluctuations of the incoming photon intensity. The energy resolution was set at 180 meV. The photon energy axes were calibrated using the Ti L_3,2_ and O K absorption edge features of a rutile TiO_2_ single crystal. The incident photon beam was at normal incidence to the sample surface with the linear polarization vector parallel to the c*_R_*-axis of the VO_2_ films. For the XAS studies, the samples were measured at room temperature and at an elevated temperature of 400 K.

## 3. Results and Discussion

### 3.1. Probing Depth

**Figure 1:**
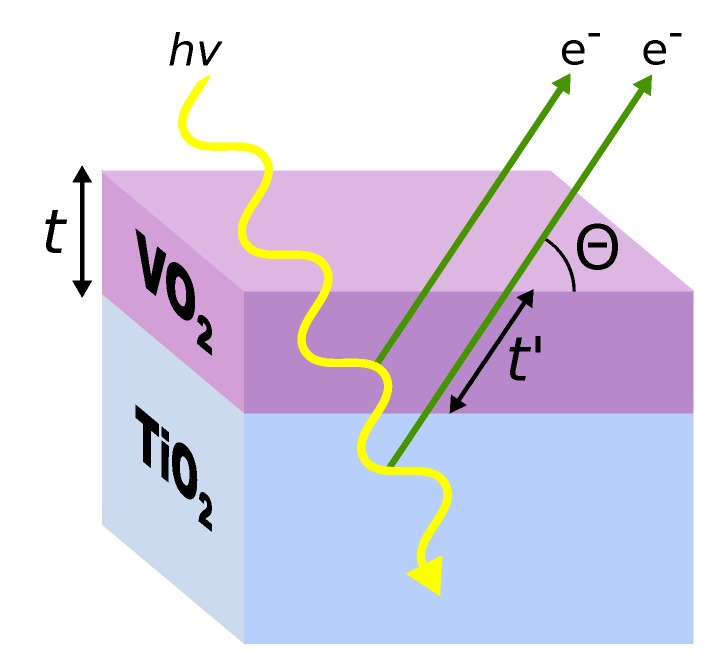
A schematic representation of our VO_2_ films of a thickness, t, grown epitaxially on our TiO_2_ (100) substrates. The photoelectrons, once generated, must travel through some length of the material before they escape the surface and can be detected.

Both HAXPES and XAS (in TEY mode) depend on photon-in, electron-out processes. A schematic of this general process is shown in Figure 1. The photoelectrons, once generated, must travel through some length of the material before they escape the surface and can be detected either with an electron analyzer (HAXPES) or from the drain current (TEY XAS). Typical escape lengths are of the order of nanometers or less, although they are highly dependent on the kinetic energy of the electron. For films with a thickness of only a few nanometers, it should be anticipated that the detected electrons will have originated from both the film and substrate beneath.

#### 3.1.1. HAXPES

**Figure 2:**
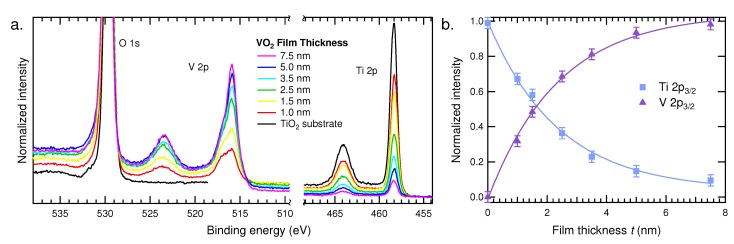
(**a**) X-ray photoelectron spectroscopy (HAXPES) spectra collected from six VO_2_/TiO_2_ (100) thin films with thicknesses varying from 7.5 to 1 nm; (**b**) normalized HAXPES signal intensity of the V 2p_3/2_ and Ti 2p_3/2_ *versus* film thickness.

Figure 2a shows the HAXPES spectra of the O 1s, V 2p and Ti 2p core levels collected from six thin film samples with varying film thickness, as well as a rutile TiO_2_ single-crystal substrate for reference. The spectra are displayed normalized to the O 1s peak intensity for clarity, since both oxide materials contain two oxygens per cation. The O 1s shows a single peak near 530 eV, while the V and Ti 2p core levels each manifest as doublet peaks due to spin-orbit splitting. The Ti 2p peaks are each symmetric, indicating a single Ti^4+^ oxidation state in all samples. The V 2p peaks, however, show a shoulder on the high binding energy side, indicating some surface oxidation has likely occurred in each film. The thickness of each film is reflected by the relative signal intensity from the V 2p and Ti 2p core lines. The thickest film of 7.5 nm shows a strong V 2p signal, while most of the signal originating from the substrate is attenuated by the film. On the other end, the thinnest film of 1 nm shows a very weak V 2p signal, while the corresponding Ti 2p peaks are only slightly diminished as compared to the bare substrate.

Peak fitting was carried out to obtain quantitative depth resolved information. All fits were performed using a Shirley background and Voigt line shapes consistent with Silversmit *et al.* [56]. The Ti 2p region was peak fit using two peaks representing the Ti 2p_3/2_ and 2p_1/2_. The O 1s and V 2p core levels were fit concurrently using a single peak for the O 1s and two peaks for each spin-orbit split V 2p peak with separation of 1.15 eV representing V^4+^ and V^5+^ contributions to the overall intensity. The total normalized intensity of the Ti 2p_3/2_ and V 2p_3/2_ peak fits, respectively, are plotted in Figure 2b as a function of film thickness. These peak fits were used to examine the interfacial quality of these films.

In the hard X-ray regime, the emitted photoelectrons have sufficiently large kinetic energy, such that the forward scattering is the dominant interaction with the solid, thus we can ignore the effects of elastic scattering [47]. This means that the profile of escaping electrons should follow an exponential decay with the distance the electron must travel in order to escape the solid. Because of this, the intensity of a single photoelectron peak, which is proportional to the number of photoelectrons reaching the detector, $I^{d}$ can be represented as:

(1) $$I^{d}=I^{0}\int_{0}^{\infty }e^{\frac{-z}{\lambda }}dz$$

where $I^{0}$ is the unattenuated intensity at the point of origin, *z* is the depth from the sample surface and *λ* is the effective attenuation length [57]. Referring to Figure 1, if a perfect interface is assumed for a film of known thickness *t*, photoelectrons can traverse a maximum length of $t^{\prime }=t/\sin\theta$ through the film, where *θ* is the angle of photoelectron emission relative to the sample surface (take-off angle). Thus, the signal originating from the film is given by:

(2) $$I_{f}^{d}\propto 1-e^{\frac{-t}{\lambda \sin\theta }}$$

Similarly, the signal originating from the substrate is:

(3) $$I_{s}^{d}\propto e^{\frac{-t}{\lambda \sin\theta }}$$

These functions were used to fit the data for varying *t* shown in Figure 2b, where in this case, the V 2p_3/2_ signal represents the film and Ti 2p_3/2_ is the substrate intensity. Both fits independently yield 1/*λ* = 0.42 ± 0.03 nm^−1^, corresponding to an effective attenuation length of 2.38 nm. This means that $\lambda_{\text{V}{\text{O}}_{2}}\approx \lambda_{\text{Ti}{\text{O}}_{2}}$, which is to be expected due to the close binding energies of the 2p core levels and similarities between these materials, e.g., structure, density and atomic weight. Alternatively, depth-resolved information can be gained by varying *θ* for a given film thickness in principle; however, in this case, *θ* is fixed to maximize our probing depth.

Agreement with these exponential fits requires two things: that the nominal film thicknesses are accurate and that the interfaces are reasonably abrupt. Fortunately, the thicknesses are often well-calibrated from the *in situ* REED oscillations during MBE growth, as they are in this case. This means that this approach is a convenient non-destructive procedure to investigate the quality of the interface in contrast to the more typical techniques, e.g., sputter depth profiling and cross-sectional transmission electron microscopy (TEM).

**Figure 3:**
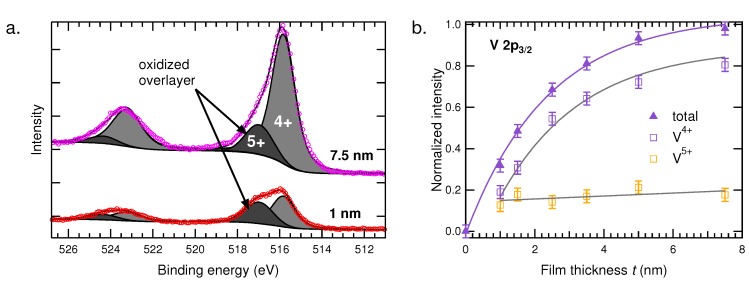
(**a**) HAXPES spectra collected from the 7.5 and 1 nm films. Peak fits show the contributions of V^4+^ from the film and V^5+^ from the overlayer; (**b**) normalized HAXPES signal intensity of the V 2p_3/2_ showing the V^4+^ and the V^5+^ contributions separately *versus* film thickness.

We now turn our attention to the oxidized surface. So far, we have considered the entire thickness of the film, *i.e.*, both the expected V^4+^ and the V^5+^ from the oxidized overlayer. When considered separately, the two contributions behave very differently as a function of film thickness. Figure 3a shows the peak fitting for the 7.5 nm and 1 nm samples. The V^4+^ peak is much larger for the thicker 7.5 nm film, while the V^5+^ peak intensities are comparable.

Figure 3b shows the V^4+^ and V^5+^ contributions separately for the whole sample set. From this, it can be observed that the the V^5+^ is roughly constant across each sample, revealing that there is no correlation with film thickness. This indicates that the the naturally-forming oxidized layer has likely reached its limiting thickness [58]. In contrast, the V^4+^ can be fit with the same exponential function as the total V 2p_3/2_ intensity, although rigidly lowered by a constant value. This is due to the overlayer attenuating the V^4+^ signal, and because it is of similar thickness on each sample, it does not significantly effect the exponential character. Additionally, from the V^5+^/V^4+^ ratios and knowledge of *λ*, we can also estimate the inherent thickness of the oxidized overlayer on our air-exposed samples. Here, we find an average value of ∼0.35 nm, although we do not expect an atomically sharp interface between the overlayer and film. This thickness notably should be considered as a correction to each nominal film thickness, since a small portion of the stoichiometric VO_2_ film is lost to this oxidation.

**Figure 4:**
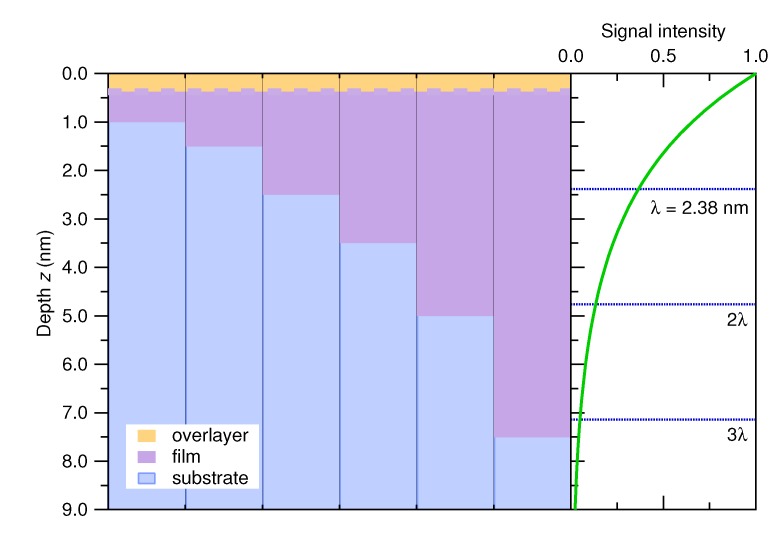
A summary of the depth-resolved HAXPES analysis of the six VO_2_/TiO_2_ (100) thin films. To the right is the exponential profile of the HAXPES probing depth for comparison.

A summary of the depth-resolved analysis from HAXPES is shown in Figure 4. Each sample shows the ∼0.35-nm oxidized overlayer (orange) on top of the VO_2_ epilayer (purple) of different thickness with an abrupt interface to the TiO_2_ substrate (blue). To the right, the experimentally-determined intensity profile is shown, *i.e.*, $e^{-z/\lambda }$, where *λ* = 2.38 nm. One advantage of HAXPES, compared to the more conventional use of soft X-rays is not just the maximum depth that can be probed, but the fact that for the thinner samples, the entire film can be probed within the first two *λ*, which corresponds to 87% of the total signal. This means that all of the regions of interest, *i.e.*, overlayer, film and substrate, can contribute significantly to the collected spectra.

#### 3.1.2. XAS

**Figure 5:**
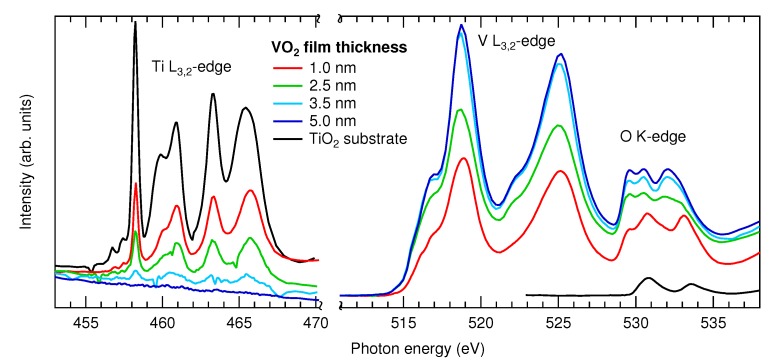
Ti L_3,2_-, V L_3,2_- and O K-edge XAS spectra of four VO_2_/TiO_2_ (100) films of different thickness and a TiO_2_ single crystal for reference.

Figure 5 shows the XAS spectra of the Ti L_3,2_-, V L_3,2_- and O K-edges of four VO_2_ films of different thickness as compared to a TiO_2_ substrate. The V L_3,2_-edge, which has a turn on at ∼515 eV, and the O K-edge at ∼529 eV are near enough in energy that they can be measured easily in a single spectra. XAS in the TEY mode is considered surface sensitive, since it is dominated by inelastically-scattered low-energy electrons. The exact probing depth is difficult to quantify, but the effective escape depth of these electrons is estimated to be below 5 nm for XAS in the soft X-ray range [59]. With our methodology, it is simple to verify this experimentally. The intensity variation of the Ti and V L-edges follows a qualitatively similar trend to the Ti and V 2p core lines in the HAXPES due to the different sample thicknesses. Here, the signal from the Ti L-edge can no longer be observed for the 5 nm-thick film, and the V L-edge has reached a maximum intensity. This shows that the maximum probing depth is indeed less than 5 nm for these films. For films below this thickness, the O K-edge should contain both information from the oxide film and the oxide substrate originating from beneath, making interpretation somewhat difficult.

### 3.2. Temperature-Dependent Electronic Structure

As VO_2_ films approach nanoscale dimensions, various exotic phases have been predicted, such as half metallicity and semi-Dirac cones [60,61]. With the interface quality established and the surface well-characterized, we can now confidently study the effects of film thickness on the thermally-induced MIT. To investigate the temperature dependence of the electronic structure, we compare the 5-nm and 1-nm VO_2_ films. The 5-nm film is of sufficient thickness to avoid spectral contamination in the XAS, while the HAXPES can still probe the entire film effectively.

**Figure 6:**
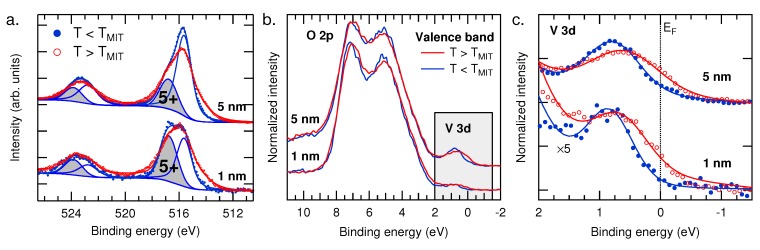
(**a**) V 2p HAXPES measured above (red) and below (blue) T_*MIT*_ for two VO_2_/TiO_2_ (100) films with thickness of 5 nm and 1 nm, respectively. Peak fitting results are shown for the low temperature measurement; (**b**) Valence band HAXPES; and (**c**) an expanded view of the topmost V 3d states of the same two samples.

Figure 6a shows temperature-dependent changes to the V 2p core level HAXPES spectra for the 5-nm and 1-nm VO_2_ films. The resulting peak fits are shown for the low temperature (T < T_*MIT*_) insulating phase. This is to highlight that the only significant spectral difference between the two films is the difference in the relative intensity of the V^5+^/V^4+^ peaks expected from the different VO_2_ film volumes being sampled. Despite these differences, the high temperature (T > T_*MIT*_) measurements display clear changes from the low temperature spectra of each film. Both the 5-nm and 1-nm films develop a similar shoulder structure on the lower binding energy side of each of the V 2p peaks. This shoulder is due to the screening channel only present in the metallic state, as observed by Eguchi *et al.* [41]

Perhaps the most direct spectroscopic measure of the MIT is in the valence band HAXPES spectra. The valence band consists of a broad O 2p-derived band from ∼2 to 9 eV and a distinct V 3d feature near the Fermi level (E_*F*_). Figure 6b shows the valence band HAXPES normalized to the O 2p band for the two films, with an expanded view of the V 3d states shown in Figure 6c. Below T_*MIT*_, the 5-nm film displays the V 3d as a clear peak just below 1 eV. In contrast, when measured above T_*MIT*_, there is a pronounced increase of spectral weight at E_*F*_. This is consistent with the changes associated with the MIT observed in thick (∼40 nm) films also grown on TiO_2_ (100) substrates [31]. For our 1-nm film, similar changes with temperature are observed, although the signal from the V 3d is much weaker. Notably, these spectra are shown multiplied by five times after normalization to the O 2p band. This is because of the O 2p contribution from the TiO_2_ substrate to the valence band spectra. The comparable intensity to the 5-nm film after this multiplication further attests to the accuracy of the film thickness. This confirms that the electronic transition is not disrupted, even at a film thickness of 1 nm.

**Figure 7:**
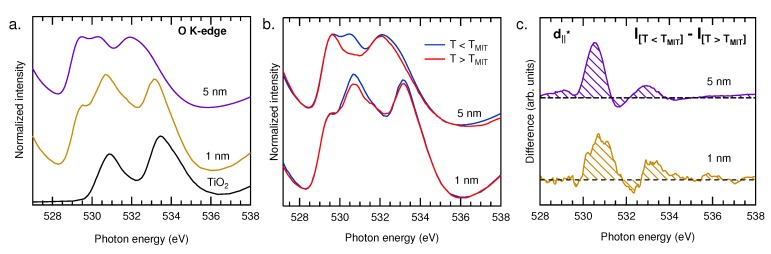
(**a**) O K-edge XAS from two VO_2_/TiO_2_ (100) films with thicknesses of 5 and 1 nm and a TiO_2_ single-crystal reference; (**b**) O K-edge XAS from the same two VO_2_/TiO_2_ (100) films measured above (red) and below (blue) T_*MIT*_; (**c**) intensity difference between high and low temperature O K-edge measurements for the two films.

The O K-edge XAS spectra below T_*MIT*_ of these two films are shown in Figure 7. In Figure 7a, the spectra are compared to the O K-edge of a bare TiO_2_ substrate. The 5-nm film shows three features corresponding to the unoccupied π* band at ∼529.5 eV, the d_||_* band located just above the π* and the broader σ* band at ∼532 eV. The presence of the d_||_* feature confirms that there are indeed V-V dimers in the 5-nm film in the low temperature insulating phase, as expected. The 1-nm film shows clear spectral mixing from VO_2_ and TiO_2_. From this spectra alone, it is not possible to discern any clear evidence of a d_||_* feature, especially due to the overlapping TiO_2_ feature at 531 eV.

To investigate whether the V-V dimers persist in thin films down to 1 nm, we look to the temperature-dependent changes shown in Figure 7b. After careful normalization, the 1-nm film does appear to show spectral changes from low to high temperature similar to that on the 5-nm film, where the d_||_ vanishes completely for T >T_*MIT*_. Since the O K-edge of TiO_2_ does not show any changes with temperature, the only changes must be associated with the VO_2_ film. Figure 7c shows the difference spectra of the O K-edge measurements, *i.e.*, $I_{\left[T<T_{MIT}\right]}-I_{\left[T>T_{MIT}\right]}$. From this, it is clear that the 5-nm film and 1-nm film show near identical orbital changes with temperature. This spectral signature confirms that even for the 1-nm film, the V-V dimers are formed in the insulating phase, consistent with the monoclinic M1 crystal structure, and vanish at high temperatures, consistent with the rutile crystal structure.

## 4. Conclusions

We have presented in this paper HAXPES and XAS studies of ultra-thin oxide/oxide heteroepitaxial films. A convenient, non-destructive methodology to characterize the film surface and interface and to study the phase-change behavior of correlated oxide materials is described. Here, this methodology is used to demonstrate that high quality VO_2_/TiO_2_ (100) films with sharp interfaces display spectral signatures of the MIT for films as thin as 1 nm. For all film thicknesses (7.5–1 nm), we observe a low temperature insulating phase containing V-V dimers and a high temperature metallic phase with no evidence of dimers. Our findings demonstrate that the intrinsic properties of the MIT for VO_2_/TiO_2_ (100) remain unchanged even for less than 1 nm of material (equivalent to about two unit cells for this orientation).
